# Stimuli-Responsive, Cell-Mediated Drug Delivery Systems: Engineering Smart Cellular Vehicles for Precision Therapeutics

**DOI:** 10.3390/pharmaceutics17081082

**Published:** 2025-08-21

**Authors:** Samson Sitheni Mashele

**Affiliations:** Centre for Quality of Health and Living, Faculty of Health and Environmental Sciences, Central University of Technology, Free Sate, Bloemfontein 9301, South Africa; smashele@cut.ac.za; Tel.: +27-51-5073111

**Keywords:** cell-mediated delivery, stimuli-responsive, smart drug delivery, erythrocytes, immune cells, exosomes, stem cells, precision therapeutics, controlled release

## Abstract

Stimuli-responsive, cell-mediated drug delivery systems represent a dynamic interface between biological functionality and engineered control. Leveraging the inherent targeting properties of erythrocytes, immune cells, stem cells, and exosomes, these systems offer a promising strategy for precise therapeutic delivery. In this review, we provide a comprehensive analysis of the design principles and biological underpinnings of stimuli-responsive carriers that release payloads in response to endogenous triggers (e.g., pH, redox, enzymatic activity) or external stimuli (e.g., light, ultrasound, magnetic fields). We further examine current strategies for loading and functionalizing cellular carriers, highlight key therapeutic applications across oncology and regenerative medicine, and assess translational progress and regulatory challenges. This review underscores the emerging clinical potential of intelligent cell-based delivery vehicles and outlines future directions for their optimization and implementation.

## 1. Introduction

Stimuli-responsive, cell-mediated drug delivery systems have revolutionized targeted cancer therapy over the past decade. Utilizing the inherent homing ability and biocompatibility of cellular carriers, such as erythrocytes, immune cells, stem cells, and exosomes, these platforms protect therapeutic payloads and precisely deliver them to pathological sites. Yet, challenges remain in controlling drug release, imaging, and achieving clinical translation [1].

The field of drug delivery has long pursued solutions to the perennial challenges of therapeutic efficacy and safety. Conventional delivery platforms, including liposomes, micelles, and polymeric nanoparticles, have made strides in improving solubility and circulation times but often fall short when it comes to tissue specificity, biocompatibility, and controlled release. A major limitation lies in their inability to discriminate adequately between healthy and diseased tissues, leading to systemic toxicity and suboptimal drug localization [1,2]. Furthermore, rapid clearance by the mononuclear phagocyte system, poor penetration across biological barriers, and instability in circulation exacerbate the limitations of synthetic systems [3].

In contrast, cell-mediated delivery systems present a compelling alternative by harnessing the innate properties of biological cells. These systems leverage the biocompatibility, targeting ability, and long circulation half-lives of cells, such as erythrocytes, immune cells, stem cells, and exosomes. Unlike synthetic carriers, these living vectors can actively home to disease sites in response to chemotactic cues, traverse complex biological barriers, and engage in dynamic interactions with the host microenvironment [4,5]. Red blood cells (RBCs), for instance, can be engineered to encapsulate or attach drugs and therapeutic enzymes, benefiting from their natural stealth characteristics and prolonged lifespan in circulation [6]. Similarly, immune cells, such as macrophages and T-cells, have been exploited for their inherent ability to infiltrate tumors and inflamed tissues, serving as Trojan horses for therapeutic delivery [7].

Among stem cells, mesenchymal stem cells (MSCs) have garnered considerable attention due to their tumor-tropic behavior and immunomodulatory properties, making them suitable for delivering anticancer agents and regenerative therapies [8]. Exosomes, the nanoscale extracellular vesicles secreted by these and other cell types, provide another promising avenue. These vesicles naturally transport proteins, lipids, and nucleic acids between cells and are now being engineered to carry exogenous therapeutic payloads with high precision [9]. Their nanoscale size, low immunogenicity, and intrinsic targeting capabilities position them at the frontier of nanomedicine.

However, while cell-mediated systems offer improved biocompatibility and targeting, the challenge of uncontrolled or premature drug release remains a significant barrier. This has driven interest in stimuli-responsive strategies, which integrate internal or external triggers into the delivery vehicle’s design. These smart systems are programmed to release their payload in response to specific pathological cues, such as acidic pH, redox gradients, or enzyme overexpression, or in response to externally applied stimuli, like light, ultrasound, or magnetic fields [9,10,11]. For example, pH-sensitive systems take advantage of the acidic microenvironment of tumors or intracellular compartments to achieve site-specific drug release [10]. Enzyme-responsive platforms are designed to respond to matrix metalloproteinases (MMPs) or other disease-specific enzymes, while redox-sensitive carriers release their contents in response to elevated levels of reactive oxygen species or intracellular glutathione in cancer cells [11]. A recent study by Thirumalai et al. demonstrated a pH-responsive nanoformulation using oxidized sodium alginate derivatives loaded with 5-fluorouracil, which enhanced anticancer efficacy through controlled release and diketone tautomer stabilization [12].

Externally triggered systems offer an additional layer of control. Near-infrared light can be used to induce photothermal effects or activate photoresponsive linkers, allowing for spatial and temporal control over drug release. Similarly, ultrasound facilitates the permeabilization of cell membranes through sonoporation and can trigger the release of payloads from acoustic-sensitive carriers. Magnetic fields, when used with magnetically labeled cells or nanoparticles, can both guide carriers to target sites and induce hyperthermic effects for synergistic therapy [13,14,15].

Despite the promise of these advanced platforms, integrating stimulus responsiveness into cell-based systems introduces new complexities. Maintaining cell viability, functionality, and carrier integrity post-engineering remains a delicate balance. Nevertheless, the integration of stimuli-responsive mechanisms with cell-mediated delivery systems opens a new paradigm in precision medicine—one that unites the adaptability of biological carriers with the controllability of engineered stimuli.

The novelty of this review lies in systematically integrating multi-modal stimuli—including pH, enzymatic, redox, light, ultrasound, and magnetic field triggers—within cell–hybrid systems currently progressing toward clinical translation. Unlike previous reviews focusing on either cell carriers or synthetic nanomaterials, our work offers a unified framework, bridging advanced design strategies with theranostic functionalities and real-world applicability.

In this review, we aim to report the current landscape of stimuli-responsive, cell-mediated drug delivery systems, emphasizing hybrid platforms that merge controlled release, imaging capability, and clinical feasibility. We present design principles, comparative analyses, preclinical performance, and regulatory challenges confronting this growing field. We begin by categorizing the various types of cell vehicles, followed by a detailed examination of stimuli-responsive mechanisms and how they are incorporated into living carriers. We then explore techniques for cargo loading and functionalization, analyze therapeutic payloads, and assess translational progress, including preclinical and clinical developments. Finally, we discuss the overarching challenges—technical, biological, and regulatory—that must be overcome to bring these next-generation systems to clinical reality.

## 2. Cell-Mediated Delivery Vehicles

The use of living cells as delivery vectors provides an elegant approach to targeting and releasing therapeutic agents. This section discusses the four main types of cell-based carriers—erythrocytes, immune cells, mesenchymal stem cells (MSCs), and exosomes—along with hybrid vesicle systems that combine properties from different cell types or synthetic materials.

### 2.1. Erythrocytes

Red blood cells (RBCs) represent one of the earliest and most extensively studied cellular carriers for drug delivery. Their innate properties—long circulation half-life, immune tolerance, and lack of nucleus and organelles—make them ideal for accommodating a wide variety of therapeutic agents. Typically, RBCs are loaded with drugs using techniques like hypotonic dialysis, electroporation, or encapsulation during erythropoiesis. These methods allow for the entrapment of enzymes, chemotherapeutics, or small molecules within the cytosol, enabling prolonged systemic presence and gradual drug release [14]. In addition, surface modification techniques enable the attachment of therapeutic agents or targeting ligands to the erythrocyte membrane without compromising cell viability. RBC-based systems have been explored for enzyme replacement therapy, anticancer treatments, and as decoys for toxin or pathogen sequestration. A recent study demonstrated that encapsulating L-asparaginase into RBCs significantly prolonged enzyme half-life and reduced hypersensitivity reactions in leukemia therapy [15]. Additionally, RBCs have been engineered for photothermal delivery using stimuli-responsive linkers, improving the precision of release at tumor sites [16].

### 2.2. Immune Cells

Immune cells, particularly macrophages, neutrophils, and T-cells, have gained traction as dynamic delivery vehicles due to their inherent capacity to home to sites of inflammation, infection, or malignancy. Macrophages can engulf therapeutic nanoparticles and migrate across biological barriers to deliver drugs deep within tumors or inflamed tissues. Their natural chemotactic response to cytokine gradients, such as those found in the tumor microenvironment (TME), enhances targeting efficiency. Neutrophils exhibit similar migratory behavior and have been used to deliver anti-inflammatory agents in autoimmune and injury models. T-cells, especially chimeric antigen receptor (CAR) T-cells, have been genetically modified to target specific cancer antigens and can be further engineered to carry immunostimulatory agents or nanoparticles. These strategies allow for a dual-function platform that combines immunotherapy with precise drug delivery [13,17]. Macrophage-mediated delivery of DOX-loaded liposomes was shown to enhance drug accumulation in triple-negative breast cancer models, leading to superior therapeutic efficacy [18]. Furthermore, SPION-tagged macrophages have enabled MRI-guided delivery and imaging in hepatic tumors [19]. Tumor-homing macrophages engineered for simultaneous delivery and immune activation are progressing toward clinical translation [20].

### 2.3. Stem Cells

Mesenchymal stem cells (MSCs) are multipotent stromal cells capable of differentiating into various lineages and exhibit robust tumor-homing capabilities. Their immunomodulatory properties and ability to evade host immune responses make them favorable candidates for cell-based delivery. MSCs can be loaded with therapeutic agents ex vivo and reinfused into patients, where they migrate to pathological sites, such as tumors or injury zones. Moreover, MSCs can be genetically engineered to express therapeutic genes or secrete biologically active molecules, such as cytokines or growth factors. Their use has been explored in cancer therapy, myocardial infarction, and neurodegenerative diseases [21]. However, concerns regarding their tumorigenic potential and long-term fate necessitate rigorous safety assessments. Original experimental work demonstrated that MSCs engineered to express TRAIL induced selective apoptosis in metastatic cancer cells without affecting healthy tissues [22]. Another study showed that MSC vesicles loaded with paclitaxel led to significant tumor volume reduction in ovarian cancer models [23]. Cryopreservation techniques have also been optimized to retain the functionality of engineered MSCs [24].

### 2.4. Exosomes and Hybrid Vesicles

Exosomes are small extracellular vesicles (30–150 nm) derived from the endosomal compartments of most cell types. They play a key role in intercellular communication by transporting proteins, lipids, and RNA between cells. Their natural composition, biocompatibility, and ability to cross physiological barriers like the blood–brain barrier make them ideal carriers for drug delivery. Engineered exosomes can be loaded with therapeutic cargos, such as siRNA, mRNA, or small molecule drugs, and modified with targeting ligands to enhance tissue specificity. Additionally, hybrid vesicles that combine exosomal membranes with synthetic nanoparticles have been developed to unify the advantages of both systems, such as enhanced stability, payload capacity, and targeting accuracy [25,26]. These innovations open new avenues for precision medicine, especially in oncology and neurology. Their lipid bilayer provides intrinsic stability, and their surface markers can be engineered for targeted delivery. IL-12-decorated exosomes (ExoIL-12) demonstrated prolonged tumor retention and potent antitumor effects with reduced systemic toxicity compared to recombinant cytokines [27]. In neurological applications, exosomes released by GDNF-transfected macrophages successfully delivered therapeutic proteins across the blood–brain barrier in Parkinson’s models [19]. Hybrid vesicles combine natural membranes with synthetic nanocarriers to create multifunctional systems with improved targeting and payload capacity. A study in *Science Advances* described exosome–nanoparticle hybrids that exhibited improved circulation and tumor accumulation [28]. Another study detailed SPION-loaded exosomal constructs for theranostic applications, enabling both delivery and magnetic imaging [29]. These systems present a flexible platform for integrating multiple functional modalities in one carrier.

Table 1 summarizes the critical attributes of major cell-based carriers used in stimuli-responsive drug delivery. While erythrocytes provide long-term circulation and excellent biocompatibility [30], their functionalization capacity is limited. In contrast, immune cells and MSCs offer active tropism toward pathological tissues but may suffer from immunogenicity or tumorigenic concerns [8,31]. Exosomes, as natural nanocarriers, cross biological barriers effectively but face scalability and stability issues [32].

## 3. Stimuli-Responsive Strategies

The design of drug delivery systems that can respond intelligently to specific physiological or externally applied stimuli represents a major advancement in precision medicine. When merged with the unique capabilities of cell-based carriers, stimuli-responsive mechanisms can significantly improve the spatiotemporal control of therapeutic release, enhancing both efficacy and safety. These systems can be broadly classified based on the origin of the triggering stimulus: internal, deriving from the disease microenvironment, and external, administered intentionally from outside of the body. Each approach offers distinct advantages and engineering considerations.

Internal stimuli-responsive strategies exploit the altered biochemical milieu typical of pathological sites. For example, pH-responsive mechanisms have been widely studied in cancer therapy due to the acidic microenvironment of solid tumors and intracellular compartments, such as endosomes and lysosomes. Drug-loaded erythrocytes or exosomes can be functionalized with acid-labile linkers that degrade under low pH conditions, thus facilitating localized release within tumor tissues or following endocytic uptake [19,20].

Another internal cue is the redox gradient observed between the extracellular matrix and the intracellular cytosol, particularly in tumor cells that exhibit elevated levels of glutathione (GSH). Delivery systems can exploit this difference by incorporating disulfide bonds or other redox-sensitive linkages into their structural backbone. Upon cell internalization, the higher GSH concentration cleaves these linkages, resulting in controlled drug liberation. This approach has been effectively applied to stem-cell-derived vesicles and immune cell conjugates [21].

Enzyme-responsive systems represent a third internal modality. Matrix metalloproteinases (MMPs), cathepsins, and other disease-associated enzymes are often upregulated in tumors, inflamed tissues, and sites of infection. Carriers can be engineered with peptide linkers that are cleaved by these enzymes, enabling release specifically within affected tissues [22]. Such strategies have proven effective in both animal models of cancer and inflammation, where MMP-sensitive immune cell carriers achieved superior tumor penetration and therapeutic effect.

In addition, reactive oxygen species (ROS)-responsive designs have gained traction due to the elevated oxidative stress in many pathological states. Chemical moieties, such as thioketals and boronic esters, are oxidized in the presence of ROS, disrupting carrier integrity and triggering payload release. Exosome-based delivery systems have been developed with ROS-sensitive modifications, targeting inflamed or neoplastic tissues where ROS concentrations are pathologically high [23].

Complementing internal stimuli, external triggers offer the advantage of user-controlled, on-demand release. Light-responsive systems, particularly those activated by near-infrared (NIR) light, provide precise spatial control with minimal tissue damage. Carriers incorporating NIR-sensitive linkers or photothermal agents, such as gold nanorods, can be administered systemically and activated locally at the target site. This strategy has been used in exosome and stem-cell-derived delivery platforms, where laser irradiation induces localized heating and controlled drug release [24,25].

Ultrasound-responsive carriers utilize acoustic waves to enhance tissue penetration and disrupt carrier membranes. Nanobubbles or microbubbles conjugated to cellular vehicles can cavitate under ultrasound exposure, releasing encapsulated drugs in deep tissues. This technique has proven especially beneficial for targeting dense tumor matrices or crossing the blood–brain barrier [26].

Magnetically responsive systems, on the other hand, involve the use of magnetic nanoparticles that are guided or activated by an external magnetic field. Superparamagnetic iron oxide nanoparticles (SPIONs) are commonly used for this purpose. They can be incorporated into macrophages or stem cells, which are then navigated to the target tissue and subjected to alternating magnetic fields to induce localized hyperthermia and trigger drug release [27].

Thermal-responsive systems exploit phase transitions in thermosensitive polymers or lipids that occur at mildly elevated temperatures (typically 40–45 °C). These transitions can be induced externally via photothermal conversion or magnetic heating. When used in conjunction with cell-based carriers, such as neutrophils or T-cells, these systems provide a robust method for spatiotemporal control of therapeutic release [19].

Together, these stimuli-responsive strategies enable cell-mediated delivery platforms to dynamically respond to physiological cues or physician-directed interventions. By integrating environmental sensing with precise actuation, these systems represent a critical leap forward in the development of intelligent therapeutics tailored to the complexities of human disease. The next section will discuss how therapeutic agents are loaded into these smart carriers and how their release kinetics are fine-tuned for clinical use.

**Figure 1:**
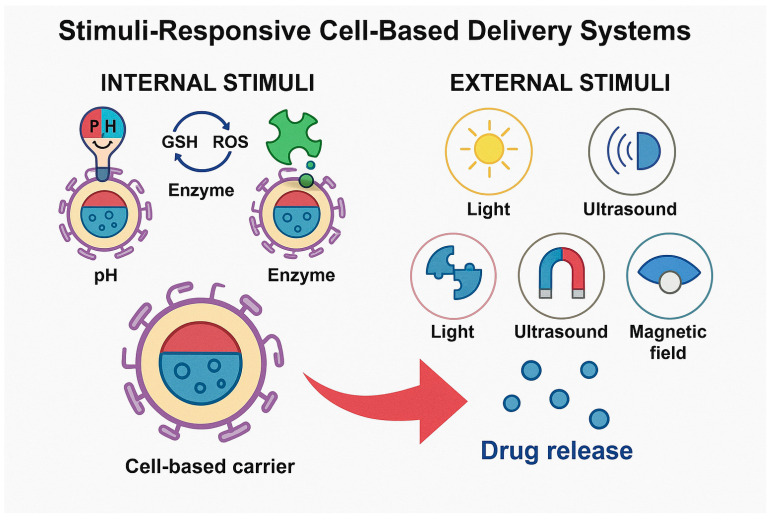
Schematic illustration of internal versus external stimuli-responsive mechanisms in cell-mediated drug delivery systems.

Figure 1 visually differentiates between internal and external stimuli that trigger drug release from cell-based carriers. Internal stimuli include pH shifts, redox gradients, and enzyme expression typical of tumor or inflamed tissues. External stimuli involve externally applied cues, such as light, ultrasound, magnetic fields, or heat. These signals activate engineered release mechanisms within or around carriers like erythrocytes, immune cells, MSCs, or exosomes, enabling spatiotemporally controlled drug release. This dual classification highlights the versatility of smart delivery systems in achieving precision therapeutics.

## 4. Loading and Functionalization Techniques

A crucial aspect of developing cell-mediated delivery systems lies in the effective incorporation of therapeutic payloads and the subsequent functionalization of the carrier to enhance targeting and release performance. The strategies used must balance several competing factors, including cargo stability, cellular viability, and release kinetics. Approaches vary based on the type of cellular carrier employed, the physicochemical nature of the drug, and the intended site of action.

Loading techniques can be broadly categorized into physical, chemical, and biological methods. Physical methods include electroporation, sonication, and hypotonic swelling, all of which transiently permeabilize the cell membrane to facilitate drug entry. For example, electroporation uses brief electric pulses to create nanopores in the membrane, allowing for the intracellular incorporation of small molecules, nucleic acids, or proteins. While effective, these methods must be carefully optimized to preserve cell viability and function [29].

Chemical strategies often involve the conjugation of drugs or nanoparticles to the cell surface using cleavable linkers or receptor–ligand interactions. Covalent binding through bioconjugation techniques, such as click chemistry or thiol–maleimide coupling, can ensure stable drug attachment while allowing for controlled release in response to specific stimuli, such as pH or enzymatic activity. These approaches are particularly advantageous for immune cells and stem cells, which may otherwise internalize the therapeutic cargo inefficiently [30].

Biological methods utilize the natural uptake mechanisms of cells, such as phagocytosis or receptor-mediated endocytosis. Macrophages, for instance, can be incubated with nanoparticle formulations that are readily engulfed and retained intracellularly. Similarly, stem cells can internalize exogenously supplied therapeutic agents or genetic material via endocytosis, especially when preconditioned with specific ligands or engineered vectors [31].

Beyond loading, functionalization refers to the addition of targeting moieties, stealth features, or stimuli-responsive elements. Surface functionalization can involve coating the carrier with ligands that enhance homing to specific tissues, such as peptides that bind integrins in tumor vasculature or antibodies that recognize overexpressed receptors in diseased tissues. Polyethylene glycol (PEG) is often used to reduce immunogenicity and extend circulation time [33]. While PEGylation improves nanoparticle stability and prolongs systemic circulation, it can paradoxically hinder therapeutic efficacy by reducing cellular uptake. The polyethylene glycol (PEG) chains form a steric barrier that shields drug carriers from immune recognition and premature clearance, yet this same barrier may limit endocytosis by target cells due to diminished receptor interaction. Additionally, repeated administration of PEGylated agents can induce anti-PEG antibodies, potentially accelerating clearance and compromising delivery efficiency [1]. These trade-offs necessitate optimized PEG density and length to balance stealth and target engagement.

Recent advancements have also enabled the genetic engineering of cells to express surface markers or secrete therapeutic proteins upon reaching the target site. CAR-T cells are a prime example of this strategy, where T-cells are modified to express chimeric antigen receptors that recognize and bind to cancer cell antigens. These genetically modified cells can be further equipped with drug payloads or biosensors to create multifunctional delivery platforms [31].

Importantly, the choice of loading and functionalization strategy must be tailored to the carrier type and clinical application. Techniques must be validated not only for efficiency and stability but also for their impact on the carrier’s viability, homing ability, and immunogenicity. Standardized protocols and quality control measures are essential for scaling these technologies for translational and clinical use.

The following section explores the diverse range of therapeutic payloads that can be delivered using these functionalized cell carriers, including small molecules, biologics, nucleic acids, and theranostic agents.

## 5. Therapeutic Payloads

The versatility of cell-mediated delivery systems is exemplified by their ability to transport a broad spectrum of therapeutic payloads. These include small molecule drugs, proteins and enzymes, nucleic acids, and theranostic agents. The compatibility of various payloads with specific cellular carriers, coupled with the integration of stimuli-responsive features, enables highly targeted and context-specific therapeutic interventions [32].

### 5.1. Small Molecule Drugs

Small molecules remain a cornerstone of pharmacological therapy across numerous disease areas, including oncology, infectious diseases, and inflammatory conditions. Their incorporation into cell carriers can enhance bioavailability, reduce systemic toxicity, and enable site-specific accumulation. For example, doxorubicin-loaded macrophages have been shown to home to tumor sites and release the chemotherapeutic agent in response to tumor-specific stimuli, thereby maximizing local efficacy while sparing healthy tissues [32]. Similarly, mesenchymal stem cells loaded with paclitaxel or camptothecin have demonstrated improved anti-tumor activity and reduced adverse effects in preclinical models [34].

### 5.2. Proteins and Enzymes

Therapeutic proteins and enzymes, such as cytokines, growth factors, and replacement enzymes, benefit from cell-mediated delivery by overcoming their inherent instability and rapid degradation in systemic circulation. Red blood cells have been employed to encapsulate enzymes like asparaginase for leukemia therapy, extending enzyme half-life and reducing immunogenicity [35]. Immune cells and stem cells have also been engineered to secrete therapeutic cytokines or express enzyme prodrugs that become activated at target sites, offering controlled and sustained protein delivery.

### 5.3. Nucleic Acids

Cell-based systems are uniquely suited to deliver nucleic acid therapeutics, including plasmid DNA, mRNA, siRNA, and CRISPR-Cas9 components. These payloads can be internalized via electroporation or transfection techniques and released in response to specific intracellular cues. Exosomes, due to their ability to naturally carry and protect RNA molecules, are particularly attractive for gene therapy applications. Engineered exosomes have been used to deliver siRNA to silence oncogenes or inflammatory mediators in target cells with high specificity and minimal off-target effects [36].

### 5.4. Theranostic Agents

The convergence of therapy and diagnostics—theranostics—has become increasingly important for real-time monitoring of treatment efficacy and disease progression. Cell-mediated systems can co-deliver therapeutic agents and imaging contrast materials, such as fluorescent dyes, magnetic nanoparticles, or radionuclides. For instance, macrophages loaded with superparamagnetic iron oxide nanoparticles and doxorubicin can be tracked via magnetic resonance imaging (MRI) while simultaneously delivering cytotoxic drugs to tumors [26]. This dual functionality enhances personalized medicine approaches by allowing clinicians to adjust treatment based on live feedback.

The integration of imaging modalities, such as fluorescence and photoacoustic agents, into stimuli-responsive carriers has significantly advanced the theranostic potential of cell-mediated delivery systems. Fluorescence imaging—owing to its high sensitivity and compatibility with near-infrared dyes—enables real-time tracking of drug distribution and carrier biodistribution. However, it suffers from limited tissue penetration. To address this, photoacoustic imaging combines optical contrast with ultrasound resolution, allowing for deeper tissue visualization and functional imaging of tumor microenvironments. For example, exosomes loaded with near-infrared fluorescent dyes or conjugated to indocyanine green (ICG) have been successfully employed for tumor imaging and image-guided therapy [1]. Similarly, macrophage-based delivery of photoacoustic probes has demonstrated precise accumulation in hypoxic tumor regions, aiding in guided intervention [2]. These advances underscore the synergistic potential of combining responsive drug release with diagnostic feedback in a single carrier.

Each type of payload presents unique challenges in terms of loading efficiency, stability, and controlled release. However, advances in carrier engineering and stimuli-responsive designs continue to expand the range and functionality of payloads that can be effectively delivered using cell-based systems. The next section will discuss the progress made in translating these platforms from laboratory research to clinical application.

## 6. Translational and Clinical Applications

Despite remarkable progress in preclinical studies, the translation of cell-mediated delivery systems into clinical practice remains an ongoing challenge. However, several platforms have advanced to early-phase clinical trials, and a few have shown promise in late-stage development. The successful clinical translation of these systems hinges on demonstrating scalability, reproducibility, safety, and regulatory compliance.

Erythrocyte-based carriers have the longest track record in clinical translation. Several RBC-encapsulated formulations, such as GR-ASPA (asparaginase-loaded RBCs) for acute lymphoblastic leukemia, have progressed to clinical trials, showcasing improved pharmacokinetics and reduced immunogenic responses compared to free drugs [37]. The prolonged circulation time and immune invisibility of RBCs allow for steady, long-lasting drug delivery, and their established manufacturing protocols facilitate clinical scalability.

Macrophage-based delivery systems are also gaining clinical traction. One notable example includes Nano-DOX macrophages—monocyte-derived cells loaded with doxorubicin nanoparticles—developed for targeted cancer therapy. These cells exploit tumor-homing behavior to deliver chemotherapeutics directly to the tumor microenvironment, minimizing systemic toxicity [38]. Similar macrophage platforms are under investigation for delivering anti-inflammatory agents in autoimmune diseases and stroke.

Mesenchymal stem cell (MSC)-based carriers have shown encouraging results in treating cancers, myocardial infarction, and neurodegenerative diseases. Trials using MSCs loaded with paclitaxel or engineered to express therapeutic genes (e.g., TRAIL, IL-12) are ongoing, with several reporting reduced tumor volumes and enhanced survival in treated patients [16]. Their innate tropism to pathological sites and immunomodulatory nature contribute to both efficacy and safety, though concerns about tumorigenicity and long-term persistence remain.

Exosome-based therapeutics are rapidly evolving, with multiple companies developing clinical-grade exosomes derived from stem cells or dendritic cells. Clinical trials have explored exosome-based delivery of siRNA, immune modulators, and proteins for conditions like pancreatic cancer and graft-versus-host disease. One pioneering example is ExoIL-12, a dendritic-cell-derived exosome engineered to carry IL-12, currently in clinical testing for melanoma [39].

The clinical integration of external stimuli, such as light or magnetic field activation, has also begun, particularly in oncology. Photothermal therapy combined with light-sensitive drug release is under investigation in conjunction with stem cell carriers. Magnetically responsive macrophages, tagged with superparamagnetic nanoparticles, are being explored not only for drug targeting but also as MRI-visible diagnostic agents [40].

Nonetheless, hurdles remain. Manufacturing processes must be standardized to produce consistent, high-purity cell carriers under Good Manufacturing Practices (GMP). Regulatory guidelines for complex cell-based products, particularly those involving genetic modifications or synthetic enhancements, are still evolving. Safety concerns, such as immunogenicity, off-target effects, and long-term biodistribution, must be meticulously addressed in human studies.

Despite these barriers, the future of cell-mediated, stimuli-responsive systems in clinical therapeutics is promising. The convergence of biotechnology, materials science, and clinical pharmacology is fostering the emergence of highly tailored, intelligent therapies that move beyond conventional pharmacological paradigms. Table 2 summarizes the development stage, carrier types, therapeutic agents, clinical status and target diseases. The next section explores the current challenges and future directions needed to realize the full clinical potential of these advanced delivery systems.

## 7. Challenges and Future Perspectives

While the potential of stimuli-responsive, cell-mediated delivery systems is immense, several critical challenges must be overcome to enable their widespread clinical application. These challenges span the technological, biological, and regulatory domains and are key considerations for the next phase of development and translation.

One of the foremost technological barriers is the issue of scalability. Current methods for loading and functionalizing cell carriers are often manual, low-throughput, and difficult to standardize. For instance, electroporation and incubation techniques, while effective in laboratory settings, require optimization for industrial-scale processing under Good Manufacturing Practices (GMP) without compromising cell viability or function. Automated microfluidic systems and bioreactor-based expansion of cellular carriers are being explored to address this gap [43].

Maintaining the functional integrity and homogeneity of engineered carriers is another concern. Even minor variations in the source of primary cells, culture conditions, or surface modifications can significantly impact carrier behavior, biodistribution, and therapeutic efficacy. This variability can pose challenges for reproducibility across clinical batches and study centers. It also complicates the pharmacokinetic and pharmacodynamic profiling essential for regulatory approval [44].

From a biological perspective, concerns regarding immune interactions, off-target effects, and long-term biodistribution persist. Although cell carriers generally exhibit low immunogenicity, modifications, such as PEGylation, nanoparticle loading, or genetic editing, can alter their immunological footprint. Furthermore, carrier cells’ fate after delivery, such as apoptosis, phagocytosis, or differentiation, remains unpredictable and must be carefully monitored to ensure safety [45].

Another key challenge is regulatory complexity. These advanced systems straddle multiple regulatory categories, combining features of biologics, devices, and drug delivery platforms. Regulatory frameworks are evolving but still lack harmonized guidelines for combination products that include genetically modified or hybrid cellular components. Clear definitions around potency assays, release criteria, and long-term follow-up protocols are essential to guide safe development [46].

Looking ahead, several opportunities promise to propel the field forward. Synthetic biology and CRISPR technologies could allow for precise programming of cells to autonomously respond to pathological signals. AI-driven modeling of delivery dynamics and patient-specific biomarker profiling may enable personalized cell delivery regimens. Moreover, integration with wearable or implantable devices could allow for real-time control over stimuli-triggered release, ushering in a new era of closed-loop therapeutic systems [47].

**Figure 2:**
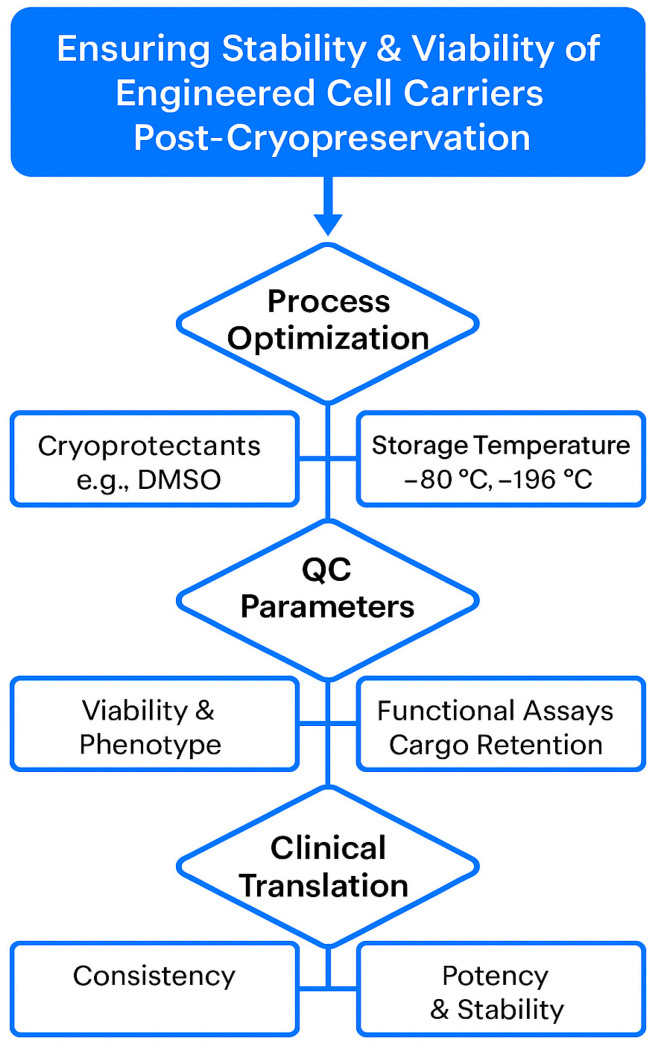
Illustrates the standardized workflow and critical checkpoints necessary for maintaining the long-term stability, viability, and functionality of engineered cell carriers intended for clinical use.

One of the critical bottlenecks in the clinical translation of cell-mediated drug delivery systems is ensuring the long-term stability and viability of the engineered carriers. For therapeutic use, these systems must retain function over extended storage and withstand transport without compromising drug integrity or cellular behavior. Cryopreservation remains the most viable method for large-scale biobanking, but challenges persist—especially for complex constructs like drug-loaded mesenchymal stem cells (MSCs) or exosomes. Cryoprotective agents (e.g., DMSO) may impact membrane integrity or interfere with drug payload stability. Moreover, repeated freeze–thaw cycles can compromise the targeting ability and release profiles of carriers. Emerging strategies, including vitrification, lyophilization of exosome formulations, and controlled-rate freezing protocols, show promise in preserving cell function and therapeutic potency [48]. Nonetheless, standardizing quality control metrics and Good Manufacturing Practice (GMP)-compliant storage processes will be essential for clinical deployment. Figure 2 illustrates the standardized workflow and critical checkpoints necessary for maintaining the long-term stability, viability, and functionality of engineered cell carriers intended for clinical use. It also underscores the integrated approach required to ensure consistent, safe, and effective delivery systems from lab bench to bedside.

Collaborations between bioengineers, clinicians, and regulatory bodies will be crucial in navigating these challenges. Establishing open-source standards for design, validation, and quality assurance can accelerate progress. As knowledge accumulates and technologies mature, stimuli-responsive, cell-mediated delivery systems are poised to become a central pillar in precision medicine.

## 8. Conclusions

Stimuli-responsive, cell-mediated drug delivery systems represent a remarkable convergence of biology, engineering, and clinical science. By integrating the navigational intelligence and biocompatibility of living cells with smart release mechanisms responsive to internal or external cues, these systems offer unprecedented precision in therapeutic delivery. Their capacity to deliver a wide array of payloads—including small molecules, proteins, nucleic acids, and imaging agents—further enhances their adaptability across diverse disease domains [49].

The progress made in recent years demonstrates a clear trajectory from proof-of-concept studies to early-stage clinical applications. Platforms based on erythrocytes, immune cells, stem cells, and exosomes have shown distinct advantages, and the integration of stimuli-responsive triggers continues to expand their functionality and specificity. However, challenges in scalability, standardization, safety, and regulation must be systematically addressed to fully realize their clinical potential.

Continued interdisciplinary collaboration and innovation will be vital in overcoming these barriers. As new tools in synthetic biology, nanotechnology, and computational modeling become integrated into delivery system design, we anticipate the emergence of highly personalized, adaptive therapies capable of responding dynamically to the biological environment. These next-generation systems stand to transform the landscape of medicine, offering safer, more effective treatments tailored to the complexities of individual patients and diseases.

## Figures and Tables

**Table 1:** Comparison of cell-based carriers for stimuli-responsive drug delivery.

Carrier Type	Size (nm)	Circulatory Lifespan	Tropism	Advantages	Limitations
Erythrocytes	6000–8000	~120 days	Passive, long circulation	High biocompatibility, long half-life	Limited functionalization options
Immune Cells (e.g., Macrophages)	10,000–12,000	Few days to weeks	Inflammation and tumor targeting	Natural homing, immunomodulation	Rapid clearance, immune activation
Mesenchymal Stem Cells (MSCs)	15,000–20,000	Several days	Tumor, ischemic, inflamed tissues	Regenerative potential, immune privilege	Tumorigenic potential, differentiation capacity
Exosomes	30–150	Minutes to hours	Broad biodistribution, limited specificity	Nanoscale size, cross biological barriers	Short half-life, batch heterogeneity

**Table 2:** Clinical status of cell-mediated drug delivery systems.

Cell Carrier	Therapeutic Agent	Indication	Clinical Status	Reference
Erythrocytes (RBCs)	L-asparaginase (GR-ASPA)	Acute lymphoblastic leukemia	Phase II trials	[41]
Macrophages	Doxorubicin-loaded liposomes (Nano-DOX)	Triple-negative breast cancer	Preclinical/investigational	[27]
MSCs	Paclitaxel, TRAIL, IL-12	Ovarian cancer, melanoma	Early-phase clinical/preclinical	[42]
Exosomes	IL-12 (ExoIL-12)	Melanoma	Clinical (NCT01550523)	[19]
Exosomes	siRNA (engineered)	Pancreatic cancer	Investigational	[29]
Cell Carrier	Target Disease		Targeting Mechanism	
MSCs	Glioblastoma		CXCR4–SDF1 axis	
Macrophages	Breast cancer		Chemokine receptor-mediated homing	
Exosomes	Pancreatic cancer		Surface protein recognition (CD47, integrins)	
RBCs	Leukemia		Passive targeting via long circulation	
